# Varieties of dispositional essentialism about natural laws

**DOI:** 10.1007/s13194-021-00375-4

**Published:** 2021-07-23

**Authors:** Salim Hirèche

**Affiliations:** Université de Genève, Geneva, Switzerland

**Keywords:** Dispositional essentialism, Laws of nature, Natural necessity, Natural self-governance

## Abstract

An important task for metaphysicians and philosophers of science is to account for laws of nature – in particular, how they distinguish themselves from ‘mere’ regularities, and the modal force they are endowed with, ‘natural necessity’. Dispositional essentialism about laws (for short: ‘essentialism’) is roughly the view that laws distinguish themselves by being grounded in the essences of natural entities (e.g. kinds, properties). This paper does not primarily concern how essentialism compares to its main rivals – Humeanism and Armstrongeanism. Rather, it distinguishes and comparatively assesses various brands of essentialism – which mainly differ as to where exactly they take laws to find their essentialist *sources* (e.g. in particular entities, like electrons, or in larger pluralities of entities, or in the world as a whole), and what they take to be the *targets* of laws, namely what they apply to. Yet, this internal comparison is not unrelated to the more general debate about laws: the main criteria with which I compare these essentialist views concern how they can deal with some of the main objections faced by essentialism in general (the modal status it typically attributes to laws, which some think is too strong; and its alleged incapacity to account for the most 'general' laws, like conservation laws), and how they can keep what is arguably the main intuitive advantage of essentialism over its rivals (the fact that, on this view, things “govern themselves”). Thus, the paper also concerns the relative position of essentialism in the larger debate about laws – ultimately bringing support to it.

## Introduction

One major task for metaphysicians and philosophers of science is to account for laws of nature – in particular, their specificity with respect to other regularities, and their modal force, known as ‘nomic’ or ‘natural’ necessity. There are three dominant families of views about laws of nature.^1^ On broadly *Humean* accounts (after Hume, 1740), laws are contingent generalizations that distinguish themselves by meeting some (epistemic or practical) criteria – simplicity, strength and balance, on the notorious “best-system” account (Lewis, 1973). On what I will call *Armstrongeanism*, laws are generalizations that correspond to relations of “contingent necessitation” between relevant universals (Armstrong, 1983; Dretske, 1977; Tooley, 1977). For a large part of the last century, those two approaches dominated philosophical debates on laws. It is only a few decades ago that *dispositional essentialism* (for short: essentialism) emerged as a new approach (Harré & Madden, 1975; Shoemaker, 1980; Swoyer, 1982; Ellis, 2001; Molnar, 2003; Mumford, 2004; Lowe, 2006; Bird, 2007a; Heil, 2012). On this view, laws distinguish themselves from other generalizations by being true in virtue of the essences of natural entities (e.g. kinds, properties).^2^

In this paper, I will not primarily be concerned with how essentialism compares to its main rivals. Rather, I want to distinguish and comparatively assess various brands of essentialism – which mainly differ as to where exactly they take laws to find their essentialist *sources* (e.g. in particular entities, like electrons, or in larger pluralities of entities, or in the world as a whole), and what they take to be the *targets* of laws, namely what they apply to. However, this comparison within essentialism will not be unrelated to the more general debate about laws: the main criteria that I will rely on to assess these brands of essentialism concern how they can deal with some of the main objections faced by essentialism in general (the modal status it typically attributes to laws, which some think is too strong; and its alleged incapacity to account for the most 'general' laws, like conservation laws), and how they can keep what is arguably the main intuitive advantage of essentialism over its rivals (the fact that, on this view, things “govern themselves”). Thus, by arguing that a certain kind of view within the essentialist family is particularly well fitted to meet these crucial challenges, I will also, more generally, bring support to essentialism as a candidate approach to laws of nature.

The structure of the paper is as follows. In the next part (§2), I first present essentialism as one of the main three approaches to laws of nature, pointing out some of its main potential advantages and disadvantages with respect to its rivals. In this light, I then suggest three criteria to comparatively assess the various brands of essentialism to be considered: extensional accuracy; modal status of the laws; ability to preserve the intuition that things govern themselves. In part §3, based on those criteria, I compare two views which differ as to where they take laws to find their essentialist source – in the world as a whole (“global essentialism”) or in more specific entities within the world (“local essentialism”). In part §4, I argue that, with respect to the criteria considered, what crucially matters to a comparative assessment, beyond the essentialist source of laws, is their target (what they apply to), especially the relation between their source and target. Accordingly, I consider a view that I call “coordinated essentialism”, on which every law has the same entities (whether local or global) as both its source and target. My main conclusions will be that, if one is to be an essentialist at all, one should probably be a local essentialist rather than a global one, and anyway a coordinated essentialist rather than a non-coordinated one.

## The dispositional essentialist approach to laws of nature

### Dispositional essentialism and its main rivals

Before considering different brands of dispositional essentialism, I will quickly describe the context of the larger debate about laws of nature. Beyond setting up the general background for the discussion to come in later sections, the main purpose of this brief and partial overview is to identify some of the main features that distinguish essentialism from its main rivals (whether potential advantages or disadvantages), as those should in turn be particularly relevant to a comparative assessment within essentialism – the idea being that the best form of essentialism should be the one that can best deal with the objections against this approach, and preserve its main advantages. As suggested earlier, taking those criteria as a basis, my internal comparative assessment of different brands of essentialism will ultimately also be relevant to an external comparative assessment of essentialism and its non-essentialist rivals: if it can be shown that a particular view within the essentialist family does particularly well *in those respects*, which are crucial to essentialism’s relative position in the larger debate about laws, it also brings support to essentialism more generally.

First of all, the dispositional essentialist approach distinguishes itself from its main two rivals by being based on a radically different fundamental ontology: one where (at least some of the) fundamental entities are dispositions, or powers^3^ – as opposed to categorical properties.^4^ There is a huge and still ongoing debate on Humean *versus* dispositional metaphysics, both having their alleged virtues and drawbacks. On the one hand, with fundamental entities as dispositions, dispositionalists can account for causation, laws and other modal phenomena without having to postulate mere possible worlds; modality, as it were, is already “contained” in the actual world. Moreover, if fundamental entities are dispositional, it makes it easier for us to know them, at least assuming that what we have epistemic access to is the dispositional or causal behaviour of fundamental entities – e.g. electrons having the disposition to repel each other when in proximity. Yet, dispositions, precisely because they can be seen as “modal” entities, have faced various attacks, like the notorious charge of “meinongeanism”, to the effect that such entities lack “reality” (see Armstrong 1997: 79–80; Bird, 2007b; Lowe, 2006). Accordingly, categoricalism – the ontological background for both Humeanism and Armstrongeanism – avoids those problems. However, by having all fundamental entities as purely categorical, it faces difficulties when it comes to determining the identity of those entities. First, a metaphysical issue: their identity may be doomed to be purely “quidditistic”, a mere “thisness” (see Armstrong, 1989; Bird, 2005a; Kistler, 2002). For instance, unlike dispositionalists, categoricalists deny that electrons have their dispositional properties (e.g. the disposition to repel other negatively charged particles) as part of their identity; on categoricalism, in another possible world, electrons might have had a different dispositional behaviour (e.g. the actual behaviour of protons), while still being electrons. Yet – the objection goes on –, if dispositions are excluded from electrons’ identity, it seems difficult to say what this identity consists in – except the brute, trivial property of being electrons (a “quiddity”). Second, categoricalism faces a related epistemic issue: assuming that all we have epistemic access to is dispositional (all we can know is the causal roles, not what plays those roles), but fundamental properties are purely categorical, we are doomed to “epistemic humility”, a position of radical of ignorance about the fundamental level of the world (e.g. Shoemaker, 1980: 215; Lewis, 2001: 204, 211).

What will be of more direct interest to us is how, on the basis of those different fundamental ontologies, the three dominant views considered here account for laws. Three important, and somehow related, problems are the *distinction* problem (How do laws distinguish themselves from mere regularities?), the *explanation* problem (How can laws explain the corresponding regularities?), and the *modal status* problem (What is the modal force that laws seem to be endowed with?). As I said earlier, on Humeanism, laws distinguish themselves from other regularities with mainly epistemic, or practical, criteria – as Lewis recognized, whether those criteria give us the “real” laws of nature depends on whether nature is kind to us (see Lewis, 1973: 74; Lewis, 1994: 479). Thus, there seems to be no “robust”, metaphysical difference between laws and other regularities. As regards explanation, on the Humean picture, the regularities that we observe in the world are not to be explained by laws – or by anything else. Those regularities, and more generally the distribution of fundamental properties in spacetime, are to be accepted as brute. Finally, the modal force that laws seem to be endowed with is only apparent according to Humeanism: in the last analysis, laws are as contingent as other regularities.

Arguably, those three features of Humeanism are at odds with widely shared pre-theoretical intuitions about laws. It seems that laws do distinguish themselves from other regularities in a metaphysically robust way: there is something in the world that makes the claim that electrons repel when in proximity significantly different from the claim that there is no gold sphere with a diameter greater than 17 kms. It also seems that laws do explain the corresponding generalizations: electrons repel each other in the whole spacetime because it is a law of nature. Finally, laws do seem to have some form of necessity attached to them: electrons do not *happen* to consistently repel each other; there is a sense in which they *have* to – while there is no sense in which gold spheres *have* to be less than 17 kms in diameter.

Amstrongeanism differs from Humeanism in those three important respects. It does not face the distinction problem, at least not in the same way: laws involve a second-order ‘contingent necessitation’ relation between the relevant universals, whereas mere regularities do not. Laws are also supposed to explain the corresponding regularities: the fact that electrons and protons attract each other everywhere in spacetime does not have to be accepted as brute – it is explained by the corresponding law of nature, in the form of a relation between the universals involved. Finally, Amstrongeanism can account for the intuition that there is a modal force attached to laws: although laws are not metaphysically necessary, they are not simply contingent either; they have a *sui generis* modal force – what Armstrong calls “contingent necessitation”. While Amstrongeanism may be taken to have an advantage in those three respects, it has faced important objections concerning both the *identification* of this *sui generis* modal force (what exactly it amounts to), and the ability of the relations of ‘contingent necessitation’ between universals to really *explain* the corresponding regularities (see van Fraassen, 1989: 96; Lewis, 1983: 366; Armstrong, 1993; Bird, 2005c).

On the three issues considered, dispositional essentialism is closer to Armstrongeanism than to Humeanism. It has a clear answer to the identification problem: laws are true in virtue of the essences of natural entities, while other true regularities are not. The essences of natural entities is also what explains the regularities corresponding to laws: electrons always repel because it is in their very nature. Finally, unlike Humeanism, essentialism can easily account for the impression that laws are endowed with a modal force: laws are metaphysically necessary, because they are essential to certain natural entities. Moreover, unlike Amstrongeanism, essentialism does not have to postulate any *sui generis* modal force (and face the corresponding difficulties). On the other hand, the strong modal force that essentialism attributes to laws is seen by many as a potential problem: on pre-theoretical intuitions, unlike mathematical theorems, laws of nature may not seem to be *metaphysically* necessary. In particular, many take counter-nomic scenarios where some natural entities do not behave according to the actual laws of nature (e.g. electrons attracting each other) to be conceivable and, correspondingly, at least *prima facie* metaphysically possible.^5^

Another core criterion to assess accounts of laws is extensional accuracy: in particular, the laws given by the account should also be laws according to our common-sense, pre-theoretical intuitions,^6^ informed by science and scientific practise – and vice versa. The main problem facing essentialism in that respect is what I call the problem of “missing entities”: on essentialism, all laws are supposed to be best understood as being true in virtue of the essence of some natural entities; yet, it has been argued that some laws (e.g. conservation and symmetry laws) are too “general” to be understood as being true in virtue of *any* specific natural entities – meaning that essentialism is extensionally incomplete (see Bigelow et al., 1992; Bird, 2005b: §7.2; Fine, 2002). It is worth pointing out here that, although the problem of missing entities has been discussed as a challenge for essentialism in particular, Armstrongeanism faces the same problem: if no particular entities (e.g. kinds, properties) can be identified as those relevant to ground a given “general” law of nature, then presumably it will also be difficult for the Armstrongean to understand that law as a relation between particular universals. As regards Humeanism, it does not seem to face the particular problem of “missing entities”, but it faces extensional problems of its own, closely related to its difficulty in resolving the distinction problem: various cases have been put forward where a law may come out as a theorem of the “best system” without being plausibly a law of nature, and vice versa (see e.g. Tooley, 1977: 669; Maudlin, 2007: 67; Effingham et al., 2010: 59–60).

A final way in which the three leading views considered here importantly differ is how they account for the widely shared intuition that the world is in some sense “dynamic”: a unified collection of things which may move, interact with each other, undergo and bring about genuine change. Arguably, Humeanism has difficulties accounting for that intuition: fundamentally, the Humean world is an atomistic mosaic of unrelated events; we may derive, at a higher level, notions of causation, movement or change from this mosaic, but fundamentally things in the world are static and epiphenomenal – nothing is *really* changing, or moving, or bringing about anything. On Armstrongeanism, things are in a sense dynamic, but not by themselves: they have to be governed “from the outside”, by laws. Yet, arguably, none of those two views really does justice to our intuitions about the world – whether our naïve, common sense picture of it, or the picture that science suggests. When an object falls on the ground as I drop it, or when two electrons repel, for instance, it looks like things being dynamic. And they seem to be dynamic by themselves: they do not need a God to manipulate them, as on older theories of the natural world; nor do they need to be manipulated from the outside by what seems to have replaced God in more recent theories, namely abstract laws à la Armstrong – relations of “contingent necessitation” which, happening to hold in this world but not in other possible worlds, are *external* to the universals that they relate. To put it otherwise, it seems that, in order to get the laws governing some given entities, all you should need is those entities themselves: just put them in any possible world, and their nomic behaviour is thereby determined – you get the laws “for free”, as it were. Dispositional essentialism seems to be able to account for this idea: on this view, things are dynamic, and they owe their behaviour entirely to themselves – the laws that things obey find their source in those things’ own natures. In a slogan, “things govern themselves”. I take this to be an important intuitive advantage of the essentialist approach.

In the light of the above general context, I will now distinguish various brands of essentialism about laws, and comparatively assess them as potential best representatives of the essentialist approach.

### Varieties of dispositional essentialism about laws

Dispositional essentialism about laws may take various forms. I take the following idea to be a minimal common ground that all existing versions of essentialism share, and that arguably all potential versions should share to count as dispositional essentialist: the laws of nature distinguish themselves by finding their sources in the (dispositional) essences^7^ of some natural entities (e.g. kinds, properties)^8^ – i.e. by being true in virtue of the essence of those entities. For instance, the Pauli Exclusion Principle (PEP) – which roughly states that no two fermions in a closed system can occupy the same quantum state at the same time – is a law of nature because it is true in virtue of the very essence of some natural entities, namely fermions (see e.g. Tahko, 2015). By contrast, the true generalization that no gold sphere is more than 17 kms in diameter is not a law because it is not true in virtue of the essence of gold, or gold spheres, or anything else. There would be different ways to formalise this basic essentialist idea, but the following simple definition will do for our present purposes:

#### Essentialism about Laws (EL)

For any plurality of natural entities xx, a generalization φ(xx) about xx is a *law of nature* iff there is a plurality of natural entities yy such that φ(xx) is true in virtue of the (dispositional) essence of yy (in symbols: ∃yy: Nyy ⋀ □_yy_ φ(xx)).^9^

The varieties of EL that I will consider mainly differ with respect to what they take to be the *sources* of the laws (the yy’s in the above definition) and their *targets*, namely what the laws apply to (the xx’s). In the example of the PEP, at least according to the above description, both the essentialist source and the target are simply fermions (understood e.g. as a natural kind). As regards the essentialist source of the laws, one main distinction to draw is between local and global entities. I will call *local* entities all the entities that are “in” the world, or constituting the world – e.g. electrons, protons, electromagnetic fields –, as opposed the *global* entity, which is the world as a whole. Local entities are in fact those that most dispositional essentialists take to be the sources of all laws. Yet, mainly as an attempt to address the problem of “missing entities” described above, some essentialists have defended the view that the most general laws, and perhaps ultimately all laws, may be grounded in the essence of the world as a whole – more precisely, in the natural kind to which the actual world belongs. This brand of essentialism was mainly defended by Bigelow et al. (1992) (see also Bird, 2005b: §7.2; Kistler, 2005: 218, fn 27). I will call *global essentialism about laws* (GEL) the strictest version of this view, on which all laws are generalizations that are essential to the world as a whole.

#### Global Essentialism about Laws (GEL)

For any plurality of natural entities xx, a generalization φ(xx) about xx is a *law of nature* iff φ(xx) is true in virtue of the (dispositional) essence of W (in symbols: □_W_ φ(xx)), where W is the natural kind to which the actual world belongs.

GEL is clearly a particular case of EL, with the additional condition that the essentialist source must be W (yy = W). For instance, on this view, the target of the PEP may still be fermions, but its essentialist source is W – the PEP is grounded in the essence of the world as a whole.

By contrast, most dispositional essentialists think that all laws can be understood as essential to local entities, without the need for a global entity like W. I will call this view *local essentialism about laws* (LEL).

#### Local Essentialism about Laws (LEL)

For any plurality of natural entities xx, a generalization φ(xx) about xx is a *law of nature* iff there is a plurality of **local** natural entities yy (i.e. **yy ≠ W**) such that φ(xx) is true in virtue of the (dispositional) essence of yy (in symbols: ∃yy: Nyy ⋀ yy ≠ W ⋀ □_yy_ φ(xx)).

When appropriate, I will also consider the merits of a hybrid view, *semi-global essentialism about laws* (SGEL), on which some laws (*general* laws, e.g. perhaps conservation laws) need to be grounded in the essence of W, while the other laws (*specific* laws, e.g. perhaps the PEP) can be grounded in the essences of local entities.

Up to now, I have distinguished forms of EL based on the essentialist source of laws. But I will also draw an important distinction based on a criterion that involves both source and target, and the relation between them. I will call *coordinated essentialism about laws* (CEL) the view that any law has some natural entities (local or global) as *both* its source and target:

#### Coordinated Essentialism about Laws (CEL)

For any plurality of natural entities xx, a generalization φ(xx) about xx is a *law of nature* iff φ(xx) is true in virtue of the (dispositional) essence of xx (in symbols: □_xx_ φ(xx)).^10^

CEL is also clearly a special case of EL (with the additional constraint that xx = yy). CEL is also what is often tacitly assumed by local essentialists – in the laws that they describe to illustrate their view, one and the same entity (e.g. electrons) often serves as both the target and essentialist source. Yet, in general, LEL does not entail CEL, no more than the other way around; they are just compatible – and the same goes for GEL and CEL.

Now, on what basis should we comparatively assess those brands of essentialism? As noted above, in the context of the more general debate about laws of nature described in §2.1, the most relevant criteria should have to do with the main distinguishing features of essentialism (whether its alleged problems or virtues). Some of those features will be less relevant to a comparison, because they will presumably be shared by any of the versions of essentialism distinguished above, and indeed any potential version of EL – for instance, all will rely on a metaphysical background including fundamental dispositional entities, and all will explain the lawful regularities in the world in essentialist terms.

By contrast, the following three criteria do not only play a crucial role in the general debate about essentialism and its rivals; they are also most relevant to a comparison between the various brands of essentialism distinguished above, as we will see. The first criterion is *extensional accuracy*. This obviously includes the requirement that the true generalizations coming out as laws on the theory should indeed be those that are laws, and vice versa – according to our common-sense, pre-theoretical intuitions, informed by science and scientific practise. In that respect, the problem of “missing entities”, supposed to pose a threat to essentialism, will be of particular interest. Yet, this is not enough: to be extensionally correct, a theory should also correctly describe the targets and sources of laws. In particular, it will not do, to ensure completeness, to invoke any arbitrary entity as the essentialist source of a given law: the entity should clearly be relevant and plausible as the source of that law – again in the light of pre-theoretical, scientifically informed, intuitions.

The second criterion concerns the *modal status* of laws, another crucial respect in which essentialism seems to distinguish itself from its rivals. On the one hand, many think that laws are not metaphysically necessary; on the other hand, essentialism is supposed to have the consequence that laws are metaphysical necessary – as we will see, things are in fact not that simple once we distinguish finer-grained forms of essentialism. The third criterion that we will use concerns the idea of *self-governance*. As I said, essentialism seems to be well-suited to a picture of the world where things “govern themselves”, and I take this to be an intuitive advantage of this approach. Again, as we will see when drawing finer-grained distinctions within essentialism, not all essentialist views can preserve this advantage to the same extent. In sum, I will rely on the following three criteria:

1. ***Extensional correctness***: the generalizations that are laws according to the theory should include all and only the generalizations that are laws on pre-theoretical, scientifically informed, intuitions; moreover, the way laws are described on the theory – in particular, their proposed sources and targets – should fit our pre-theoretical, scientifically informed, intuitions.
2. ***Modal Status***: the theory should attribute the right modal status to laws of nature.
3. ***Self-Governance***: the theory should preserve the key essentialist notion that things govern themselves.

With this in place, we can now turn to our comparative assessment. Here is the plan for the remaining sections. In Section 3, I will focus on the source of laws, considering local essentialism (LEL) and global essentialism (GEL) – as well as semi-global essentialism (SGEL). In §4, the focus will be on the relation between the source and target of laws. I will show that, for the criteria considered – extensional accuracy, modal status, and self-governance –, what mostly matters, in fact, is whether essentialism takes the sources and targets to be coordinated or not, whatever those happen to be (local or global). Accordingly, I will consider coordinated essentialism (CEL), and argue that it is plausibly the best form of essentialism in those respects.

## The source of laws: local *versus* global essentialism

The main advantage that GEL is supposed to have over LEL concerns extensional accuracy (1), in particular completeness (the problem of “missing entities”). But the main proponents of GEL also claim an advantage with respect to modal status (2): GEL is supposed to avoid LEL’s consequence that laws are metaphysically necessary. In what follows, I question those two claims, and also argue that GEL may not be able to preserve the idea that things govern themselves (3).

### Extensional accuracy: ‘general’ laws and the problem of ‘missing entities’

Common, local essentialism may be quite natural for various laws of nature: for instance, the PEP is true in virtue of the essence of fermions; Coulomb’s Law is true in virtue of the essence of electrons and other charged particles; Maxwell’s equations express the essential properties of electromagnetic fields. *Prima facie*, it may seem that, ultimately, *all* laws can be naturally understood as essential to some relevant *local* entities – entities constituting the world, or “within” the world. And this is indeed what most dispositional essentialists were tacitly assuming. Yet, this assumption has been questioned. It has been argued that some laws are in some sense too “general” to plausibly find their essentialist source in any particular local entities – thereby representing a threat to LEL’s completeness. This is what I call the problem of “missing entities”.

For simplicity, I will call the allegedly problematic laws *general* laws, as opposed to *specific* laws (those that are quite naturally understood as essential to some local entities). The main candidate general laws discussed in the literature are probably conservation and symmetry laws, but examples also include universal constants and least action principles (see Bigelow et al., 1992; Fine, 2002; Bird, 2005b: §7.2; Kistler, 2005: 218). For illustration, I will focus on the example of conservation laws. For the main proponents of GEL, conservation laws indeed provide the clearest illustration of why their account of general laws has an advantage:

Conservation laws … lend themselves exceedingly eagerly to our general analysis of laws. Conservation laws do look, on the face of things, like descriptions of essential properties of the world as a whole. It takes an effort to rewrite them in such a way that they sound as though they are describing correlations of some parts of the world with others. It takes somewhat less effort, but some effort nevertheless, to rewrite conservation laws in such a way that they sound as though they are describing essential properties of mere proper parts of the world. Yet if you take conservation laws to assign essential properties to the kind of world we live in, then they can be taken more or less at face value. They fall into place without rewriting. (Bigelow et al., 1992: 386).

More generally, in the literature, conservation laws (and corresponding symmetry laws) seem to be considered as the most characteristic problematic case for LEL – the one for which a globalist solution is most plausibly needed.^11^ Yet, even for those laws, though the globalist solution may be an attractive option, it is not the only one available to the essentialist.

First, one may find their essentialist source in the essence of events, or processes. Bigelow et al. (1992) suggest that events and processes are indeed what conservation laws are about (their *target*); but they argue that they cannot constitute their essentialist *source*: “It is not essential to the category of events that they should be energy-conservative, or angular-momentum-conservative, or conservative in any other respect. Changes which were not in accordance with these conservation principles would still be events.” (Bigelow et al., 1992: 385). However, even assuming, for the sake of argument, that “event-like” entities failing to be energy-conservative are conceivable, and furthermore that this is enough to conclude that they are metaphysically possible, one may still resist the claim that those would really be events, rather than some similar, “event-like” entities. This sort of “Kripkean” move (Kripke, 1980) is a quite common one for essentialists facing such objections: for instance, an essentialist would typically argue that, even if there may be possible worlds with bodies behaving according to an inverse-cube law of gravitation, those bodies would not have mass, but some mass-like counterpart, “schmass” (see Fine, 2002, §2).

Another local entity that may (perhaps more plausibly) serve as an essentialist source for conservation laws is the very sort of conserved quantity involved. This option may also face the objection that a world where, say, the total energy of closed and isolated system is not conserved is conceivable. Yet, the same sort of Kripkean answer would be available to the essentialist. Thus, Kistler (2005: 218) argues that

it is constitutive for being the total energy-mass of a closed and isolated system to be conserved. If some energy-like quantity of such a system is not conserved, we conclude that it is just one *form* of energy, such as potential energy or kinetic energy, but not total energy. The law of the conservation of total energy … is necessary because mass-energy and other fundamental conserved quantities are conceptually linked to conservation. A property which exists in some possible world but which is not conserved is not one of them.

The legitimacy of such moves may be disputed; but it seems that it would be particularly difficult for the global essentialist to do so. This is because any essentialist, whether local or global, will presumably face analogous objections based on conceivable, allegedly counter-nomic situations; for instance, the global essentialist will face the objection that a world of the same kind as ours but with different laws (say, without the actual conservation laws) is conceivable. And it seems that the best way to answer this objection will be to deny that such a possible world would really be of the same kind as ours (unless she tries instead to deny that the situation is conceivable, or that this in turn justifies the claim that it is genuinely possible, but those alternative answers do not look very promising). It seems at least as legitimate to use this answer in the case of local entities like energy: something which would not be conserved in a closed and isolated system would be fundamentally different from the sort of thing we are used to in this world. It would not simply be total energy with some different properties, but indeed another kind of thing, with a different essence – even if perhaps “energy-like” in some sense.

A different potential problem with the localist solutions considered here, especially the one taking events or processes as the essentialist source of conservation laws, is the following: such entities – compared to the typical entities which science deals with, such as fermions, electrons or spin – may seem in some sense too “general”, or not fundamental enough. Yet, first, in the context of a debate between local and global essentialists, it seems that the latter could hardly claim to have an advantage in this respect: the world as a whole surely is a very general entity, and may not look like the typical fundamental entities that science deals with.^12^ Second, one way to avoid the problem would be to say that conservation laws find their essentialist source, not in events or processes themselves, but more precisely in the (less “general”, more fundamental) entities that constitute them – e.g. fermions, spin, and perhaps ultimately all fundamental entities. Note that, beyond conservation laws, this strategy, just like the globalist strategy, may be used as a general strategy to address *all* the alleged threats to LEL’s completeness: laws that seem too general, as they involve many local entities, perhaps all of them, can simply be understood as flowing from the essence of all those local entities, taken together. Thus, such a “collectivist” strategy, just like the globalist one, would ensure completeness; and it would do so appealing only to pluralities of familiar, local entities – without the need to introduce any unfamiliar global entity, such as the kind to which the world as a whole belongs.

### Extensional accuracy: ‘specific’ laws

I have argued that it is at best unclear whether GEL really has the advantage that it is supposed to have over LEL as regards conservation and other “general” laws: local solutions are also available, and they look at least as satisfactory. Moreover, global essentialism, in the extreme form of GEL, is not supposed to cover only the most general laws: it is also supposed to account for “specific” laws, namely those that seem to pose no threat to LEL. Indeed, the main proponents of the globalist strategy suggest that it should be generalized: “Conservation laws are not, however, the only laws which fall into place neatly, if construed as describing the natural kind to which the whole world belongs. We urge, in fact, that *all* laws of nature are best understood in this way.” (Bigelow et al., 1992: 386). Yet, there seems to be no reason to apply the globalist strategy to laws that are naturally understood as essential to some local entities – e.g. the PEP is essential to fermions. Indeed, extensional accuracy is not only about providing *some* essentialist source for each law, but about providing a plausible and natural one. In that respect, GEL seems to have a disadvantage with respect to LEL: for specific laws, not only local entities are available, but they seem to be better candidates than the global entity (at least excluding scenarios where the world is “holistic” – see Section 3.5).

An option for the global essentialist would be to use her globalist strategy only when necessary – i.e. restrict it to potential “general” laws –, while relying on local entities otherwise. This is the view I called *semi-global essentialism about laws* (SGEL). Indeed, perhaps for the reasons just mentioned, even the main proponents of the globalist strategy seem to be hesitant about adopting GEL or SGEL. Although they sometimes clearly suggest GEL, as in the passage quoted above, at other points they talk as if they were endorsing only SGEL – claiming, for instance, that Maxwell’s equations reflect the essence, not of the world as a whole, but of electromagnetic fields specifically (Bigelow, Ellis & Lierse: 383–4). SGEL may have the disadvantage of being less homogeneous. By contrast, GEL provides a very homogeneous account of laws, grounding all of them in one and the same global entity. Yet, GEL faces the objection that, for specific laws, this global entity seems unneeded, indeed inadequate – which may be more important than considerations of homogeneity. Thus, it might be that, overall, the globalist is better off adopting only SGEL.

In sum, as regards extensional accuracy, it is at best unclear whether the globalist can claim to have any advantage. For specific laws, the globalist strategy seems inadequate; and it is not even clear whether it is needed for the most general laws, as reasonable local strategies are also available, and arguably preferable. Thus, at least based on the above discussion, it seems reasonable to conclude that LEL is plausibly preferable to SGEL, and even more plausibly preferable to GEL. (Below I will briefly reconsider this comparative assessment on the assumption that the world is a “holistic” one, where local entities are in fact not (entirely) distinct and independent – see Section 3.5).

### Modal status

The main proponents of global essentialism do not only claim to have an advantage with respect to extensional accuracy – criterion (1) –, but also with respect to modal status – criterion (2). The latter advantage relies on two claims: (a) laws of nature are not necessary in the strict, metaphysical sense, but only in the (weaker) *sui generis* sense of natural necessity; and (b) although laws do come out as metaphysically necessary on the common, local brands of essentialism, they do not on global essentialism (Bigelow, Ellis & Lierse: 373–4, 387).^13^ Claim (a), that laws are not metaphysically necessary, may of course be disputed: after all, the main argument against the necessity of laws relies on the possibility of scenarios where things seem to behave counter-nomically (e.g. electrons attracting each other); yet, as we have seen, a common, “Kripkean” answer is available to the essentialist (more on this in Section 4.2). But let us assume (a) for now and focus on claim (b).

Common, local essentialism is usually taken to entail the metaphysical necessity of the laws: if, for instance, it is part of the essence of electrons that they possess (if they exist) the dispositional property of repelling other negatively charged particles, then they will have this property in any possible world (where they exist). Thus, the corresponding law of nature will be metaphysically necessary, i.e. true in all possible worlds (non-trivially so in worlds where electrons exist, and trivially so in the other ones). By contrast, on global essentialism, laws are supposed to have only a *sui generis* modal force, contained between mere contingency and strict metaphysical necessity. More precisely, this intermediate modal force is a form of *conditional* metaphysical necessity – necessity *given* this world’s belonging to the same natural kind as ours – for simplicity, we will call that kind “W”. For instance, as conservation laws are true in virtue of the essence of W, they are true, not in all possible worlds, but in those that are of kind W. This is, according to Bigelow et al. (1992), why their essentialist view avoids the consequence that laws are metaphysically necessary.

Thus, what is suggested is that laws are metaphysically contingent on global essentialism, while they are necessary on common, local essentialism. And still assuming that laws are contingent on pre-theoretical considerations – claim (a) –, the globalist has a general advantage as regards modal status. Yet, things are in fact more complicated. The first reason is that, even assuming that the laws grounded in the essences of local entities are necessary while those grounded in the essence of the global entity, W, are not, a global essentialist might not be able to claim that laws are all contingent on her view: this will depend on whether, more precisely, she holds GEL or only SGEL. In the latter case, part of the laws, the “specific” ones, will have the exact same modal status as on common LEL, namely metaphysical necessity. This is an important point because, as we have seen, the main proponents of globalism are sometimes suggesting that they hold SGEL rather than GEL – and indeed, as I have also pointed out, they may have good reasons to do so, having to do with extensional accuracy.

Moreover, besides the fact that global essentialism, as SGEL, cannot claim to avoid the consequence that (some) laws are metaphysically necessary, it would amount to a “hybrid” view of the modal status of the laws, where some laws are necessary and others are contingent. The issue would not simply be that the view is heterogeneous as regards modal status: after all, it is not that uncommon to think that different sorts of laws may have different modal forces (see e.g. Lange, 2005, 2009; Tahko, 2015). The problem is rather that, if the divide is between the most general laws, like conservation and symmetry laws, on the one hand, and the more “specific” laws on the other hand, one may rather expect that, if anything, the *former*, being more encompassing, and perhaps in some sense more fundamental, have a stronger modal status (see Lange, 2005, 2009). Yet, on SGEL, it would be the exact opposite: “specific” laws, grounded in the essence of local entities, just as on LEL, would be necessary, while conservation and other general laws, grounded in the essence of W, would come out as contingent.

Until now, I was tacitly assuming that, as the proponents of global essentialism suggest, the laws that are locally grounded are necessary, while the laws that are globally grounded are contingent – and that, consequently, whether all, some or no laws are necessary depends on whether LEL, SGEL or GEL is assumed. On the further assumption (a) that laws are in fact contingent, this implies, in particular, that GEL is preferable to SGEL, which is in turn preferable to LEL, as regards the modal status criterion (2). Yet, my second point is that the very claim that local source entails necessity and global source entails contingency is too hasty, indeed inaccurate. The modal status of laws does not only depend on whether their essentialist source is local or global: it also depends on *what* local source (if any) they are taken to have exactly, and on what *target*. On the one hand, even a law that is grounded in the essence of W may be metaphysically necessary. Indeed, this will be the case if both the source *and* the target of the law are taken to be global. The global essentialist takes conservation laws to have the whole world as their essentialist source. Those laws will then indeed be contingent *provided that* their target is taken to be some local entity like events or processes, or energy. Then, in worlds of another kind W*, conservation laws may not hold – depending on what local target we choose, this will mean that events and processes may fail to be energy conservative in such worlds, or alternatively that the total energy of closed systems in such worlds may fail to be conserved.

By contrast, still assuming that conservation laws find their essentialist source in the world as a whole, it may then seem plausible that those laws also have the whole world as their *target* – that they describe the behaviour of the world as a whole. Indeed, the main proponents of global essentialism sometimes seem to suggest such a view: when they say that laws, especially conservation and other general laws, are best understood as describing the essential properties of the world as a whole, it might suggest that what those laws apply to, the entity whose behaviour is described by them, is indeed the whole world. In any case, a view on which general laws (or perhaps even all laws) have the whole world as both their essentialist source *and* their target is an option that is available to the globalist. And on such a view, laws would be necessary, just as they are on common LEL. For if both source and target are the same global entity, W, it will not do to argue that the necessity of the law is *conditional* upon W’s existence, namely on the world’s being of kind W. That condition will trivially be met in all worlds where the target of the law exists, so that the law, describing the behaviour of that target, will be metaphysically necessary – true in all possible worlds (non-trivially so in those of kind W). Thus, contrary to what the main proponents of global essentialism suggest, attributing a global source to laws is not sufficient on its own to make them contingent.

On the other hand, conversely, LEL does not by itself entail the metaphysical necessity of the laws. LEL is only the view that laws find their essentialist sources in local entities rather than in W. But this does not yet determine *which* local entities exactly. For instance, if a local essentialist claims that a conservation law has a local entity like energy (the total energy of a closed system) as *both* its source and target, then the law will be metaphysically necessary, for the reasons just mentioned. Yet, as we have seen earlier, it is in principle also open to him to claim that the target of a conservation law is a typical local entity (energy), while their essentialist source is, say, the plurality of all fundamental entities. In this case, the law may not be metaphysically necessary: if the law is essential, not to its target, but to a larger plurality of local entities (whose existence is not entailed by the existence of the target), then there may be possible worlds in which the target exists, but the essentialist source of the law does not, so that the target is not governed by the same law anymore.

Thus, as regards modal status, global essentialism (whether as GEL or SGEL) cannot claim to have an advantage over LEL in principle, even on the assumption that laws are in fact metaphysically contingent. Things are more complex, depending on more that just whether the source of laws is local or global. Still, in practise, if we consider more specific versions of local and global essentialism, a comparative assessment is possible. In particular, if we compare a *typical* version of LEL (where every law has some entities as both source and target) to one of the most plausible interpretations of global essentialism as described by its main proponents (where all laws have a local target and a global source), then all laws will clearly be necessary on the former view, and contingent on the latter view. In this case, the globalist will indeed have an advantage on the localist as regards the modal status criterion (2) – at least on the (disputable) assumption that laws are in fact contingent.

### Self-governance

Let us now consider our criterion (3): self-governance. As it will become clear, just as for modal status, whether things fully govern themselves or not depends on more than whether the sources of laws are local or global, so that comparisons between local essentialism and global essentialism as such, without further details, are difficult to establish. Yet, for illustration, we may again consider local and global essentialism in their most typical versions – LEL with every law having some local entities as both source and target, and GEL with all laws having local targets and the global source, respectively. Even assuming that laws are in fact contingent, and that therefore globalism has an advantage over localism as regards modal status (2), this advantage will come with a disadvantage as regards self-governance (3).

For on the form of localism considered, all laws (general or specific) simply find their essentialist sources in the local entities that are also their targets. As a consequence, self-governance is clearly preserved: things govern themselves, and completely so – their behaviour comes entirely from their very nature. By contrast, on globalism, the source of the law is something over and above its target (i.e. the entities governed by the law), so that the latter is not (completely) self-governed: it is (partly) governed from the outside. Thus, the global essentialist cannot claim to have the relevant intuitive advantage over the Armstrongean anymore; at least this advantage is not so clear. For sure, even the brand of global essentialism considered here still differs from Armstrongeanism in important respects; in particular, it remains an essentialist account in that it takes laws to be true in virtue of the essence of some entity. Yet, it arguably loses some of the spirit of essentialism, which is that things essentially contain the source of their nomic behaviour, without the need for something external – whether it is “governing laws” or the essence of a global entity like W.

In sum, just as for extensional accuracy (1), it is not at all clear that globalism can claim to have an advantage over localism as regards modal status (2) and self-governance (3). In all generality, no advantage can be claimed as regards (2) because modal status depends on more than just whether the source of laws is local or global. But even assuming, for simplicity, specific versions of local and global essentialism (the most typical ones) on which all laws are necessary on the former, and contingent on the latter, the resulting advantage of the latter view as regards (2) might be compensated by a disadvantage as regards (3), because things are fully self-governing on the former view, but not on the latter. Moreover, the advantage that global essentialism may have as regards (2) relies on the assumption that laws are metaphysically contingent on pre-theoretical intuitions – a claim that, as I have pointed out, is disputable, especially from an essentialist perspective (see Section 4.2 below). Thus, overall, at least based on the above considerations about (1), (2) and (3), global essentialism does not seem more satisfactory than common, local essentialism.

### Holism?

The above discussion was based on the tacit assumption that our world is “atomistic”: roughly, that local entities (e.g. particles, fields) are distinct and largely independent from each other, and that smaller entities determine the larger entities that they compose (e.g. the plurality of all entities, the world as a whole), rather than the other way around. This assumption was particularly important when we considered ‘specific’ laws: I argued that the globalist could not apply her view to those laws (so that she could hold SGEL at most), because there was no reason why a specific law (e.g. the PEP) should be grounded in the essence of a global entity, rather than just the most directly relevant local entity (e.g. fermions). Yet, things may actually not be so simple: the world might be “holistic” instead. And if it were, this might bring some support to GEL.

Holism may take various forms. For instance, on ontological monism, the world fundamentally consists in one single entity. On a less extreme form of holism, the world can be divided into distinct local things (e.g. physical systems), but those are significantly dependent on each other and on larger wholes that contain them. Such holistic scenarios may have some plausibility. Indeed, one may find some reasons to take some of them seriously in our best current physical theories, in particular quantum mechanics, characterized by relations of entanglement (see e.g. Calosi, 2014, 2018; Ismael & Schaffer, 2020). In the context of the present discussion, the most relevant holistic scenarios to consider will be those that might favour global essentialism even for “specific” laws. I will briefly consider two.

One such scenario would indeed be ontological monism. If there is only one fundamental entity, then it seems that all laws will have to be ultimately grounded in the essence of this entity – or in the essence of the kind to which it belongs, W. What about the targets of laws? One way to go would be to say that, in such a scenario, the target of laws is also W, as the only fundamental entity. Note that, in this case, laws would preserve self-governance, and be metaphysically necessary for reasons explained above. Yet, another way to go would be to say that all laws have W as their essentialist source, but local entities as their target – as on the typical version of GEL. Local entities (e.g. fermions) may be seen as derivative entities – particular parts or “aspects” of the fundamental whole, W, that are “abstracted” from it. If laws have such local entities as their target, but W as their source, then it seems that self-governance will not be preserved, and that laws will be metaphysically contingent – as on GEL.

However, there may be another way to look at such a scenario. Laws may instead be seen as also having a local essentialist source (at least an “intermediate”, as opposed to an ultimate, local essentialist source), thereby preserving self-governance and being metaphysically necessary – as on typical LEL. For local entities, on this scenario, are not simply nomically dependent on the global entity, W (in the sense that the laws applying to them ultimately depend on W); they seem to be indeed ontologically dependent on it. Their very essence depends on the essence of W, the whole from which they are only derivative or abstracted parts. And this (intermediate) essence may be used as the source of the laws applying to them. For instance, the PEP is true in virtue in the essence of fermions – even if, ultimately, this essence is determined by the essence of W. In that sense, we preserve a form of self-governance, and the law is metaphysically necessary. For it would make no sense to say that, in another kind of world, W*, fermions would also exist but, being part of, or deriving from, a different whole W*, they would have a different essence – if they have a different essence, they *are* just not the same thing any more. In all the worlds in which fermions exist, they have the same essence (even if this essence is derived from the essence of a global entity), and they obey the same laws, which flow from this essence. Thus, even on a monist scenario, where there would be a unique global fundamental entity, W, a form of local essentialism about laws, which does not directly rely on the essence of W, may be preserved.

Let us now briefly consider a different, somewhat less extreme form of holism: fundamentally, there is not just one global entity; rather, local entities (e.g. fermions) are distinct entities in their own right, but they are highly dependent on each other. In particular, we suppose that they are *nomically* dependent on each other – in order to determine the laws governing a particular local entity, you need to fix *all* the entities that there are. Such a scenario may seem to suit global essentialism in its typical form: laws have local entities as their targets, and they cannot simply be grounded in the essences of those targets; instead, they find their source in the world as a whole, W.

A first question is whether we would really need the global entity, W, to ground the laws in such a case. After all, even if the laws applying to each plurality of entities are dependent on the plurality of all entities, we may indeed ground them in the essence of the plurality of all entities. Moreover, especially for an essentialist, who establishes a close link between the essence of a thing and its nomic or dispositional properties, it would be natural to understand the relevant dependence as *ontological* dependence: entities depend on all other entities for their very essence. Indeed, it is not uncommon for dispositional essentialists to think that fundamental entities (e.g. properties) form a web of interdependent elements, each getting its essence as a part of the structure (see e.g. Bird, 2007b). And it is also the sort of essential dependence relations between entities that the main proponents of global essentialism (Bigelow et al., 1992: 386–7) seem to suggest – except that, on their view, the essence of each entity is dependent on the essence of W as a whole, as opposed to the plurality of all entities.

At first sight, this may seem useless: what essences do we need over and above the essences of all entities, taken together? And if nothing more is needed, why introduce such a global entity on top of all the local entities? I suggest that one reason might be the following. It might be that what determines the essence of each entity, as part of the web of all entities, is not only the essences of all those entities, but the fact that those entities are *exactly* the entities that there are. In possible worlds with all the actual local entities *plus* some other entities, the whole web would be different, and the actual entities may not preserve their place in the structure. Yet, arguably, the further fact that the actual entities are exactly the entities that there are may not flow from the (derivative) essence of those entities, taken together. This might be one way to understand the essence of W, and how it brings something more than just the essence of all local entities: the essence of W contains, *in addition*, the fact that the local entities are exactly those that there actually are.

Be that as it may, whether the ultimate source of the essence of each local entity is a collective essence or a global essence, that (intermediate) essence may be used as a source for the laws that have that local entity as a target. And on such a view, just as in our first holistic scenario, a local essentialist account could still be defended. On this account, laws would be necessary, and a form of self-governance would be preserved. In sum, at least based on this very brief discussion, it is not clear that LEL would be seriously threatened, even if monism or some other form of holism turned out to hold.

## The source and target of laws: coordinated *versus* non-coordinated essentialism

### Source-target coordination and extensional accuracy

One general lesson from the above discussion is that, whether as regards extensional accuracy (1), modal status (2) or self-governance (3), what matters is not simply whether the source of laws is global or local, but also their target, and the relation between their source and target – in particular, whether those are the same or not, namely “coordinated” or not. As regards (1), beyond finding a source for each law and avoiding the problem of missing entities, what also counts, for a view of laws to be extensionally correct, is whether it attributes a plausible source to them, as well as a plausible target. As regards (2) and (3), as the above discussion already showed, whether laws are metaphysically necessary and whether self-governance is preserved clearly depend on both their sources and targets – and more specifically on whether they are coordinated or not (be they global or local). Thus, instead of focusing only on local *vs* global essentialism, it makes sense to also consider a different question – one that, to my knowledge, remains largely unexplored. Should a dispositional essentialist defend *coordinated essentialism about laws* (CEL)? In the rest of this paper, I will argue that CEL (whether local, global or semi-global) is indeed what an essentialist should adopt, based on criteria (1), (2) and (3).

Let me first briefly consider (1). As regards completeness and the “missing entities” problem, it should be noted that CEL has an advantage over both LEL and GEL: it can rely on any entities, local or global, as potential essentialist sources. On the other hand, CEL is limited by a coordination constraint: can *every* law of nature, whether ‘specific’ or ‘general’, be plausibly understood as having some entities (whether local or global) as both its source and target? The above discussion already suggested a positive answer. As regards ‘specific’ laws, a local, coordinated essentialism is clearly the best way to go as regards (1), at least excluding holistic scenarios. And even for such scenarios, I have suggested some local, coordinated solutions. (Moreover, even if we assumed that those solutions do not work for some reason, and that grounding laws in the essence of W itself is inevitable, one may still argue that the relevant laws should accordingly have W as a whole as their target, yielding a global but still coordinated solution.) As regards ‘general’ laws, we have seen that various local solutions are available. Some laws may have to be grounded in the essence of the plurality of all natural entities; and it seems that it would then be natural to also take this plurality as their target. For instance, if we take a conservation law to find its source in the essence of all local entities, as the ultimate constituents of events and processes, it seems natural to say that their target is also events and processes, and so ultimately all local entities. But I also suggested more typical localist solutions for general laws (e.g. energy as the essentialist source), and those were clearly coordinated. Thus, it may well be that no global entity is needed, even for general laws. (And again, even assuming that some general law most plausibly found its source in the essence of W, a global but coordinated solution, where the law governs the whole world, would still be available.)

Thus, even if more would be needed to properly argue that CEL meets criterion (1) (at least as well as the main other brands of essentialism), it seems at least plausible. I will now focus on (2) and (3).

### Self-governance and metaphysical necessity: a dilemma for the essentialist?

That CEL fully meets criterion (3) should be obvious: the view is tailor-made for self-governance, and it is its most salient advantage. The real issue, then, is to what extent that clear advantage may be compensated by a disadvantage as regards modal status (2). Indeed, it is not only that laws having the same entities as both their essentialist source and target *can* be metaphysically necessary, as the above discussion illustrated: CEL indeed *implies* the metaphysical necessity of all laws – just as it implies self-governance. To make it more precise, a sufficient condition for a law governing entities xx to be necessary is for it to be true in virtue of the essence of some entities yy whose existence is entailed by the existence of xx (e.g. yy is included in xx): in such a case, in all possible worlds where xx exists, yy also exists; and as it is essential to yy that the law hold if yy exists, the law will hold and govern the behaviour of xx in all possible worlds where xx exists. Thus, the law is metaphysically necessary, namely true in all possible worlds (trivially so in those where yy does not exist, which are also worlds in which xx does not exist). And this sufficient condition is trivially met for all laws on CEL, where xx = yy.

Thus, on the assumption that laws are in fact contingent, the essentialist finds himself before a dilemma: two main desiderata, full self-governance and contingency, are just incompatible. Should the essentialist still hold CEL, preserving full self-governance at the expense of contingency? I will argue for a positive answer. The essentialist is not really faced with a dilemma, because (a) the metaphysical necessity of the laws should in fact not be problematic for the essentialist, and (b) an essentialist view where laws are contingent (such as typical GEL) may indeed lead to problematic consequences.

As regards claim (a), as already pointed out, I think that the metaphysical necessity of the laws should not be such a bad consequence, especially for an essentialist. I suspect that the main reason why even some essentialists present it as a problem may be the following. The claim that the laws of nature are metaphysically necessary – i.e. the laws hold in all metaphysically possible worlds – is ambiguous between two very different readings, which are often not clearly distinguished. First, the claim may mean the following: (i) For any natural entities xx, any actual law φ(xx) governing xx also governs xx in any possible word (where xx exists); thus, φ(xx) is true in all possible worlds (trivially so in worlds where xx does not exist). This is the sense in which I used the claim that laws are necessary in this paper. Now, there is a second sense, which is sometimes suggested by Bigelow et al. (1992) (see also Kistler, 2002; Fine, 2005: 240, 242): (ii) The nomic behaviours of actual things are the same as the nomic behaviours of things in all possible worlds; one way to put it may be that there is a law φ(xx) governing some actual entities xx iff in any other possible world there is the same law φ(yy) governing some entity yy (perhaps xx = yy). Clearly, the two readings are different. In particular, to take a simple, toy illustration, a world where there is ‘schmass’, a mass-like entity behaving according to an inverse-cube law of gravitation, is metaphysically impossible if laws are metaphysically necessary in the sense of (ii), but not if they are metaphysically necessary in the sense of (i).

As I said above, the best candidate argument against (i) is based on the conceivability of allegedly counter-nomic scenarios (e.g. one with electrons attracting themselves); and even granting that such scenarios are conceivable, indeed metaphysically possible, a common Kripkean answer is available: those would not be electrons, but ‘schmeletrons’. This common answer seems perfectly legitimate in general – e.g. it is one thing to conceive two *entities* attracting each other, quite another to claim to be able to conceive specifically two *electrons* attracting each other. And as I said, an essentialist in particular, whether local or global, should accept the legitimacy of this sort of move in general, for it seems that her view will somehow rely on it as well. Thus, understood as (i), the claim that laws are necessary should not be problematic, especially for an essentialist.

On its second reading, (ii), the claim means that the set of nomic behaviours (laws) should be the same in all possible worlds. First of all, whether from a scientific or more general point of view, this use of the claim, “The laws are necessary” (or “The laws hold in all possible worlds”) is arguably less natural, and perhaps misleading: a law of nature associates a *specific entity* (or plurality of entities) with a specific behaviour, saying that if anything is (an instantiation of) this entity, it must behave in a given way – roughly, “Anything of this sort has that sort of behaviour”, rather than “There is that sort of behaviour” or “Some sort of things have that sort of behaviour”, or “Nothing has that sort of behaviour”. But let us put this issue aside to focus on the content of (ii). For instance, to use again our toy Newtonian case, (ii) excludes, not only a possible world where *mass* itself behaves according to an inverse-cube law of gravitation, but a world where *any* entity would behave according to such a law. Yet, it seems very difficult to resist the claim that such a world is conceivable. And although one may deny that conceivability *systematically* entails possibility, there seems to be no convincing reason to deny, *in this particular case*, the genuine possibility of the situation conceived – or so I will assume. Of course, the Kripkean move consisting in saying that the relevant entity is “schmass” rather than mass will be of no help here; and it is difficult to imagine what else could save (ii) from such scenarios. Thus, the claim that laws are necessary in the sense of (ii) would indeed be much more problematic and difficult to defend than (i). Yet, this can hardly be taken to be a disadvantage of local with respect to global essentialism, or a problem for CEL: *none* of the brands of essentialism considered in this paper is committed to (ii). Indeed, to my knowledge, none of the main defenders of dispositional essentialism about laws holds that claim. What they typically say, and what CEL says in particular, is that an entity could not exist in another possible world and behave according to different laws; and this is perfectly compatible with the sort of counterfactual scenarios just considered.

In sum, once we make it perfectly clear what the claim that laws are metaphysically necessary amounts to, it seems that it represent no serious threat: (i) is a reasonable consequence for an essentialist view, and the main sort of objections against it can be addressed convincingly; (ii) is much more problematic, but not a consequence of CEL.

And there might be more: an essentialist view on which laws are metaphysically contingent, such as typical GEL (laws with W as their essentialist source and local entities as their targets) may lead to problematic consequences, which a necessitarian view such as CEL avoids – this was my claim (b). More precisely, (b) relies on the two following claims: (b_1_) On a contingentist essentialist view, like typical GEL, but *not* on a necessitarian view like CEL, natural existential claims, i.e. claims that such or such natural entity (e.g. electrons) exists, have the same modal status as laws of nature. Yet, (b_2_) On pre-theoretical intuitions, natural existential claims should *not* have the same modal status as laws of nature (i.e. they should not be naturally necessary, whether natural necessity is a special case of metaphysical necessity or not).^14^ Let us consider those two claims in turn.

Earlier, I suggested that the essence of W may plausibly be understood as containing the essences of the various (fundamental) local entities, as well as the fact that those entities indeed exhaust the local entities that there are. As I said, the main proponents of global essentialism are not explicit about the exact content of this global essence, but it seems very reasonable to assume that it should at least include the fact that the world contains the natural entities that it actually contains. For if a possible world could contain no fermions, no mass, no spin, and instead contain all sorts of alien entities like ‘schmass’, and yet *still* be of the same kind as ours, W, then it would become very unclear to me what the notion of kind of world could even mean. Now, if the existence of a natural entity, say fermions, is essential to W, then the natural existential claim, p, that fermions exist will be true in all worlds of kind W. Thus, p will be metaphysically necessary *conditional* upon the world’s being of kind W. But this W-conditional metaphysical necessity is exactly the *sui generis* modal status that laws have on GEL, as we have seen. Thus, on this contingentist essentialist view, natural existential claims have the same modal status as laws (natural necessity). By contrast, on a necessitarian view like CEL, laws are simply metaphysically necessary (i.e. natural necessity is just a special case of metaphysical necessity), while natural existential claims are *not* – the property of behaving according to the PEP is part of the essence of fermions, while the property of existing is not. Thus, on CEL, natural existential claims have a different (weaker) modal status than laws of nature.

This was for claim (b_1_). Now let me briefly argue for (b_2_), the claim that, on pre-theoretical intuitions, natural existential claims do not have the same modal status as laws of nature. The general motivation for this claim may be put as follows: intuitively, there is a sense in which fermions *have* to behave according to the PEP; but it does not seem that fermions *have* to exist – anyway not in that same sense. That sense is the sense of natural necessity (whether understood as a special case of metaphysical necessity, as on CEL, or as a weaker force, as on GEL). Indeed, a common way to characterize what is natural necessary is simply as what the laws of nature entail (see e.g. Hale, 1996); and accordingly, what is naturally impossible is what the laws exclude. This view is intuitive and neutral, in that it does not depend on a particular account of what a law of nature is in the first place. And it makes it clear why, intuitively, it seems that fermions *have* to behave according to the PEP, while they do not *have* to exist in the first place. As the PEP is a law of nature, it is trivially entailed by the laws of nature. Equivalently, a situation where two fermions would occupy the same quantum state at the same time is naturally impossible, because incompatible with the PEP as a law. By contrast, it is difficult to see what law of nature would state or entail the existence of fermions. Equivalently, it is difficult to see why the non-existence of fermions (or indeed the non-existence of anything, for that matter) would be incompatible with the PEP or any other law of nature. In a nutshell, natural necessity, the modality associated with the laws of nature (however those are accounted for), concerns how the natural entities must or can behave – *not* what natural entities there are in the first place.

Let me finish by considering two rejoinders. First, one may argue that laws of nature, contrary to what I am tacitly assuming, are not telling us how certain specific entities must or cannot behave; rather, they are telling us what sorts of behaviours there must or cannot be in the world – e.g. the PEP tells us that there must exist some kind of entity such that two of them cannot occupy the same quantum state at the same time in a close system. As I said earlier, this alternative notion of a law of nature is sometimes used; and on this understanding, laws of nature would indeed entail natural existential claims. Yet, as I also said, this notion of laws seems mistaken, from both a scientific and a more general point of view. The PEP, and any law of nature, tells us how a specific entity (e.g. fermions) must or cannot behave, or more generally what properties it must or cannot have – but existence is not among those properties.

Second, one may agree that nothing in the content of laws of nature by itself entails the existence of any natural entities, but argue that such existence may still be derived from laws indirectly, based on considerations of relevance: a necessary condition for a generalization to be a law of nature is that it be relevant, i.e. governing *existing*, *natural* entities (as opposed to ‘alien’, merely possible ones). That a generalization about entities xx can only be a law of nature if xx actually exists is not something that I would deny – indeed, it follows from the definitions proposed in Section 2.2. For instance, even if it may be true that there is schmass in other possible worlds, and that it is always governed by an inverse-cube law of gravitation in those worlds, it would be strange to consider this law of schmass as a *law of nature*, precisely because it is not relevant to our natural world. Yet, all we can conclude on that basis is that the entities involved in laws *actually* exist – *not* that they *must*, as a matter of natural necessity. To get the latter claim, a further assumption would be needed, such as the following: natural necessity is subject to the S4 axiom (if p is naturally necessary, then it is naturally necessary that p is naturally necessary) – or alternatively, lawfulness is subject to an analogous iteration principle (if p is a law, then it is a law that p is a law). *Then* natural existential claims would come out as naturally necessary: if it is naturally necessary (a law) that a given generalization φ(xx) is a law, and still assuming that φ(xx)’s being a law entails the existence of xx (the relevance assumption), it indeed follows that the existence of xx is naturally necessary (a law). Yet, that further assumption about natural necessity (or lawfulness) is highly disputable (see e.g. Leech, 2016). Intuitively, natural necessity (and laws) concern the natural world; they are about first-order natural facts or generalizations – *not* second-order facts about what generalizations are naturally necessary (or laws), let alone higher-order facts.

In sum, it is not at all clear that CEL should be problematic when it comes to modal status (2), whether in general or compared to other essentialist accounts. The consequence that laws are metaphysically necessary, once the ambiguity about what it amounts to is resolved, is not that problematic, especially from an essentialist point of view – indeed, rival essentialist views on which laws are contingent, like GEL, may face resulting problems that CEL clearly avoids. Note that those considerations about modal status should also be taken into account in the comparative assessment of local and global essentialisms made in Section 3: now that the assumption that the contingency of laws is an advantage has been undermined, typical LEL (which is a special case of CEL, where every law has some local entities as both its source and target) may be (even) more clearly preferable to typical GEL.

Going back to CEL, beyond modal status (2), we have seen that it is very plausibly satisfactory (as least as much as other essentialist views) as regards extensional accuracy (1). Finally, CEL, unlike the typical (non-coordinated) form of GEL in particular, ensures that full self-governance is preserved (3). At least based on the above discussion in Sections 3 and 4, we may reasonably conclude that, if one is to be an essentialist at all about laws, then one should probably be a coordinated essentialist.

## Conclusion

In this paper, I discussed various forms of dispositional essentialism, differing with respect to what sort of entities they take to be the essentialist sources and targets of the laws. At least in the light of the above discussion – in particular Sections 3 and 4.2 –, it is reasonable to think that one should probably be a local essentialist rather than a (semi-)global essentialist – despite, in particular, alleged problems concerning completeness and modal status. But, as I argued in Section 4, a more important question to be addressed, and which (to my knowledge) has been even more largely neglected, is whether an essentialist (whether local or global or semi-global) should be a coordinated essentialist – i.e. hold the view that the essentialist sources of the laws are always the same as their targets. I argued that the answer is plausibly positive. Beyond this internal comparison, given the position of the essentialist approach in the overall debate about laws (as briefly described in Section 2), and as my assessment was based on criteria that are directly relevant to that debate, the above discussion may reasonably be taken to bring some support to the more general claim that essentialism, as coordinated essentialism, can and should remain a serious candidate account of the laws of nature.

## Footnotes

1 Though arguably the leading ones, the three approaches considered here are of course not the only available ones. In particular, while representing very different overall accounts of laws (e.g. as regards modal status), all three are *reductionist* accounts; yet, there are also primitivist views (e.g. Maudlin, 2007), and eliminativist views (for two eliminativist views based on radically different grounds (roughly, Humean and dispositionalist motivations, respectively), see Van Fraassen, 1989, and Mumford, 2005).

2 Some may prefer to reserve the term “entity” for substances, as opposed to e.g. properties. In this paper, for convenience, I use “entity” as a very broad and neutral term, also covering ontological categories such as properties and kinds.

3 Note that, among dispositionalists, some have argued for the stronger view that *all* fundamental properties are dispositional, thus adopting “pandispositionalism” (e.g. Shoemaker, 1980; Bird, 2007a). Others have defended the view that some fundamental properties are dispositional, while other ones are categorical (e.g. Ellis, 2001). Still other ones have argued that properties are neither categorical nor dispositional (in the pure, exclusive sense), but in some sense both categorical and dispositional (see e.g. Martin, 1997; Mumford, 1998; Heil, 2003). I will leave these finer-grained ontological distinctions aside in this paper – as far as I can see at least, they should not be crucial to the particular aspects of dispositional essentialist views of laws on which I will focus here (although they clearly do matter for other aspects of those views).

4 On the categorical/dispositional distinction, see e.g. Armstrong et al., 1996; Mumford, 1998.

5 For now, I will focus on this first sort of scenario (where some *actual* entities obey different laws) as a potential objection to essentialism. A second sort of allegedly counter-nomic but metaphysically possible scenario is one involving *non-actual*, or ‘alien’, entities. As I will argue in Section 4.2, the latter sort of scenario, which has also been used as an objection against necessitarian views of laws, amounts to a different objection, requiring a different sort of answer: while essentialists should accept that the first sort of scenario is indeed counter-nomic, but deny that it is metaphysically possible, they should accept that the second sort of scenario is indeed metaphysically possible, but insist that it is not counter-nomic, and perfectly compatible with essentialism.

6 By “pre-theoretical intuitions”, I mean intuitions that are (as much as possible) prior to, or not assuming, any particular metaphysical theory of laws (e.g. Humeanism or essentialism).

7 I put “dispositional” in brackets to remain neutral on whether *all* laws can be grounded in *dispositional* essences specifically. In particular, I do not exclude that (a) only *causal* laws are grounded in *dispositional* essences, and (b) at least some laws (e.g. laws of association) are non-causal (see e.g. Kistler, 2005).

8 I will be mainly interested in the question of what entities (e.g. fermions, mass, fields, all the fundamental natural entities, the world as a whole) serve as the essentialist sources and targets of laws. A *further*, ‘finer-grained’ question is what *ontological category* those entities belong to – are they e.g. properties, kinds, relations, individual objects, or tropes? Some versions of dispositional essentialism about laws operate at the level of kinds or properties, understood as Platonic universals (see e.g. Bird, 2007a; Tugby, 2013), but other versions rather rely on Aristotelian, immanent universals (Ellis, 2001; Mumford, 2004), and there are also ‘trope’ versions (e.g. Molnar, 2003) and nominalist versions (see Whittle, 2009; L. Vogt, Nominalist Dispositional Essentialism, unpublished manuscript). In this paper, I focus on the former, ‘coarse-grained’ dimension (the ‘entity’ dimension) and leave the latter, finer-grained one (the ‘ontological category’ dimension) aside. The reason for this, besides considerations of space, is that it seems to me that the latter dimension may be less crucial with respect to the three criteria that I use here for my comparative assessment. Suppose, for instance, that fermions (in the ‘coarse-grained’ sense) are both the essentialist source and the target of the PEP as a law of nature. If so, I argue, then self-governance is fully preserved for this law – whatever stance one takes on the finer-grained question. Yet, one may argue, on the contrary, that whether it is so also importantly depends on the ontological category that “fermions” is supposed to refer to. For example, on a Platonist version of essentialism, it might be that, after all, fermions are (partly) governed “from the outside”: what really does the governing is the essence of a transcendental entity (say, the Platonic property of fermionhood), which in turn can be seen as external to what is ultimately governed, namely concrete fermions (on governance and Platonist dispositional essentialism, see Tugby, 2016). This is disputable: for instance, even accepting that what is ultimately governed is individual fermions, one may reply that, on the reasonable assumption that fermions *essentially* have this transcendental property, the essence of fermions somehow ‘includes’ (perhaps as a ‘mediate’ essence in Fine’s (1995) sense) the essence of that property, so that ultimately their nomic behaviour is still fully determined by their own essence, without the need for anything external; and as a result, self-governance is fully preserved. Moreover, even assuming that there is *some* sense in which, on e.g. a Platonist version of essentialism, self-governance may not be full, or fully direct, there still seems to be a *clearer* sense in which self-governance indeed fails to be preserved when the essentialist source and target of a law differ, not simply in the fine-grained way (e.g. fermionhood as a Platonic property or kind *vs* concrete fermions), but indeed in the coarse-grained way (e.g. fermions *vs* the plurality of all natural entities, or the whole world), or when, in addition to the essence of the entities involved (fermions, or fermionhood), an external law à la Armstrong is also needed to determine the relevant nomic behaviour. Thus, in general, whether or not the ontological category dimension matters for self-governance (and the other two criteria considered here), I am inclined to think that it may matter less anyway. All this would of course require more detailed discussion and argument, which I leave hopefully for further research. For the purpose of this paper, I will talk of entities (fermions, mass, the whole natural world) loosely, ignoring finer-grained distinctions.

9 Essence here is to be understood broadly, in three respects: first, it may include, not only strict *objectual* essence (Fine, 1994), but also *generic* essence (Correia, 2006; Fine, 2015); second, it includes, not only *basic* or *constitutive* essence, but also *derivative* or *consequential* essence (see Fine, 1995; Correia, 2012); finally, it may include both *immediate* and *mediate* essence (Fine, 1995).

10 I am here taking about *entities* as the essentialist sources and targets of laws, and about the potential identity of these entities (xx = yy), in a rather loose, ‘coarse-grained’ sense (is it fermions, or electrons, or the world as a whole?). For simplicity, and although they may also play a significant role, finer-grained distinctions about ontological category (when talking about fermions, are we talking, more precisely, about fermionhood as a Platonic property, or as an Aristotelian property, or about individual fermions?) will be largely left aside in this paper – hopefully for further research (see also footnote 8 above).

11 For instance, Bird (2005b) agrees that conservation and symmetry laws are ones for which a globalist solution may be needed, while he rather suggests localist solutions for other alleged problematic cases. For instance, he seems to agree that universal constants may not, at first sight, look like they are essential to some particular local properties. Yet, he suggests that, in fact, they may not correspond to *fundamental* laws; rather, they may ultimately be reducible to fundamental laws which, in turn, would be more easily understandable as grounded in the essences of local fundamental properties (see Bird, 2005b: §7.2-(i)). He also suggests a localist answer for the least action principle. In this case, the objection is that such a principle seems to govern (“from the outside”) the evolution of a system from its initial state, rather than following from the essential properties of the initial state, as the essentialist would have it. In response, Bird (2005b: §7.2-(iii)) notes that this impression is based on the assumption that, starting from the initial state of the system, and given all its intrinsic properties, there is a multiplicity of possible evolutions or paths; then it is concluded that the least action principle is needed to explain why, among those possible evolutions, the system follows just one of them (in the case of a deterministic system). Bird questions the assumption that, given the initial state and its properties, there is a multiplicity of possible evolutions; although there is indeed a multiplicity of mathematical or logical possibilities for the evolution of the system, it may well be that the evolution “chosen” by the least action principle is in fact metaphysically necessary, and indeed flowing from the essence of the properties of the initial state.

12 On some (interpretations of) recent physical theories, in particular quantum mechanics, however, the fundamental level of the world does seem to consist in a global entity, rather than local ones – see the brief discussion of holism in Section 3.5.

13 Strictly speaking, Bigelow et.al. (1992: 373–4, 387) distinguish natural necessity from what they call “logical necessity”. Yet, I take it, the way they use this term in the context of their paper makes it quite clear that what they mean is indeed what we would now call “metaphysical necessity”, *not* logical necessity in the narrow sense of e.g. logical truth, or truth in virtue of the nature or definition of logical concepts.

14 Fine (2002: §3) suggests that natural existential claims are indeed naturally necessary – more precisely, he mainly relies on the view that *negative* natural existential claims (e.g. “There is no schmass”) are, while here I will rather be interested in positive ones. Note also that I am only talking about *unconditional* existential claims (e.g. “There are fermions”), as opposed to *conditional* ones (e.g. “If there are entities of kind K, then there are entities of kind K*”). That some of the *latter* sort of claims may be naturally necessary (i.e. entailed by laws of nature) seems plausible. Indeed, some support for this view may be found in what Kerry Mckenzie (2017, 2020) calls a “Goldilocks Principle for Fundamental Kinds” – roughly, certain symmetry principles in (standard-model) physics at least partly determine the number and types of fundamental kinds (quantum fields) that there are. As far as I can see, such a principle is best understood as entailing at most the natural necessity of *conditional* existential claims – e.g. what kinds can or must co-exist, rather than some kinds having to exist or fail to exist unconditionally.
